# Nurturing care group approach for improving animal faeces management in Ghana

**DOI:** 10.1038/s41598-025-16961-y

**Published:** 2025-10-02

**Authors:** Bismark Dwumfour-Asare, Eugene Appiah-Effah, Kwabena Biritwum Nyarko, James Ben Tidwell

**Affiliations:** 1 Department of Environmental Health & Sanitation Education, Faculty of Environment and Health Education, Akenten Appiah-Menka University of Skills Training and Entrepreneurial Development, Asante Mampong Campus, Mampong, Ghana; 2 Regional Water and Environmental Sanitation Centre Kumasi, Department of Civil Engineering, College of Engineering, Kwame Nkrumah University of Science and Technology, Kumasi, Ghana; 3 International Programs Group, World Vision US, Federal Way, Washington USA

**Keywords:** Animal faeces, Community, Livestock, Nurturing care, Sanitation, WASH, Public health, Environmental sciences, Environmental social sciences, Diseases, Risk factors

## Abstract

Animal faeces management (AFM) is key to avert zoonotic risks especially when integrated into water, sanitation, and hygiene interventions although largely neglected by conventional projects. This study focused on understanding the influence of Nurturing Care Group (NCG) approach on improving AFM in some communities within Savelugu Municipal and Sekyere East District Assemblies of Ghana. The study adopted a cross-sectional qualitative design using 12 focus group discussions and 20 in-depth-interviews with predominantly female members from 12 communities. Data analyses used inductive content analysis based on pre-structured themes from interview tools. We recorded 171 female and 4 male participants in the study. Household livestock were reared – fowls, goat, sheep, cattle and others (e.g., pets – cats, dogs, doves). The main motivation for keeping livestock was economic, nutritional and socio-cultural benefits including ritual functions, security and companionship. NCG intervention heightened awareness of safe AFM including penning, faeces composting, and other linked safe environmental sanitation practices. Barriers to uptake included lack of resources for constructing livestock shelters, lack of fodders during dry season hence scavenging, difficulty in securing veterinary services, and women inability to secure male household heads’ support on AFM decisions. Local governance structures were not fully integrated particularly in Savelugu, and even in Sekyere East, leaders’ commitment was unsustainable over time. Intervention could improve awareness on safe AFM, leading to some immediate but unsustainable behaviour change practices due to constraints that project designers should not overlook. In future, contextualized technical solutions and active involvement of key stakeholders with defined roles would be critical for sustainable uptake and impact.

## Introduction

There is adequate evidence that animals from farms, fairs, and even petting zoos could be reservoirs for enteric zoonotic pathogens for human infections via contact with animal faeces^1,2^. Animals – livestock and/ or pets, including cattle, small ruminants, fowls, dogs, and many others pose risk of infections from faecal contamination in the environment and/ or animals themselves^1,3^. Infections from animal faeces could result in nausea, vomiting, diarrhea, and sometimes severe and complicated illnesses which could lead to death^1,4^. The worst affected are mostly young children due to compromised immune systems and poorer hygienic practices such as infrequent and/ or improper handwashing, and yet more frequent hand-to-mouth behaviors^1,5^. Generally, improved health in rural settings requires a comprehensive environmental sanitation and hygiene intervention with an integrated safe animal faecal management (AFM)^6^.

Traditional water, sanitation and hygiene (WASH) interventions in developing countries do not address animal faeces management (AFM)^7^. Meanwhile, animal faeces constitute 80% of global faecal load and represent two-thirds of faecal hazards^2,6^. These are associated with domestic animal faeces than human faeces alone^7^. WASH interventions ignore AFM likely because of over-reliance on the famous traditional F-diagram that portrays strictly human-centered pathways and barriers of faecal-oral disease transmission using human faeces, fingers, flies, fields/soil, fluids, fomites, and food versus toilets, safe water, hygiene and handwashing^7,8^. This traditional model, for years, emphasizes urgency for safe human faeces management through safe WASH without any attention to AFM^7^. Meanwhile, AFM should be a major complementary component of WASH interventions for a comprehensive improved health. Current WASH interventions typically ignore that humans, animals and animal faeces share identical transmission pathways – information long visualized in the classic F-diagram^3^. In effect, upgrading WASH infrastructure without explicitly integrating AFM risks minimal health gains or, in the worst case, counterproductive outcomes^3,6^. To fill this critical gap, Penakalapati and Team in 2017 proposed a modified F-diagram that maps animal-derived transmission routes and champions a One Health approach to WASH^8^.

The concept of One Health approach to WASH postulates that “there are interconnections between the health of people, animals, and the shared environment”^6^. In traditional rural settings, completely separating livestock from human dwellings is nearly unattainable, sustaining the ubiquitous risk of zoonotic exposure via animal faeces^9^. Zoonotic-separation tools – most notably animal penning, face multiple challenges: the financial burden of round-the-clock feeding, frequent neglect of animal health and welfare, and a prevailing cultural preference for free-range husbandry^6^. Moreover, the critical livelihood purposes from livestock including supplementary income, animal-source nutrition, transport, fuel, manure for crop production, and social or ritual uses further intensify human-animal interactions and amplify the risk of zoonotic infections^6,10^.

In Ghana like other developing countries, WASH interventions including behaviour change communication approaches follow the traditional WASH programming without AFM integration. The evidence is seen in the omission of AFM in any of the environmental sanitation and WASH public policy documents including behaviour change communication strategies, guidelines, policy, programmes and plans^11–15^. Public policy directs Metropolitan, Municipal and District Assemblies (MMDAs) to enact and enforce regulations to control rearing and straying animals for protection against health hazards and sanitary nuisances^11^. Community-Led Total Sanitation (CLTS) programmes in rural and small settlements focus almost exclusively on managing human faeces^13,15^. USAID’s SPRING Ghana WASH 1,000 Strategy stands out by explicitly addressing both animal and human faecal management^16^.

Evidence on integrating AFM into WASH behaviour-change programmes in Ghana remain scarce like other developing countries. To fill this gap, World Vision launched a pilot Nurturing Care Group (NCG) project in selected rural communities. The NCG project package included: animal faecal management, household hygiene management, menstrual hygiene management, and child nutrition (including safe breastfeeding). The project used a collective action model mobilizing community members to invest measures – such as building animal shelters or pens, improving feed practices, and to improve AFM at the compound level. We conducted a qualitative evaluation assessment to explore the following key questions:

1. what aspects of the NCG approach worked or failed?
2. how has community behaviour around AFM changed?
3. which pathways exist to sustain any gains?

This paper outlines beneficiaries’ experiences with AFM under the NCG intervention. We shared an initial preprint on Research Square repository before submitting to this journal^17^.

## Brief overview of the nurturing care group (NCG) intervention in Ghana

The Nurturing Care Group (NCG) is World Vision’s (WV) adopted version of Care Group pproach (CGA) that emerged from WHO/UNICEF Nurturing Care Framework. The framework focuses on solving developmental issues such as child health with nutrition, home management, and WASH including menstrual hygiene^18^. The CGA evolved from the original Care Group model designed by Dr. Peter Ernst following the inspiration from the biblical account of Moses in Exodus 18:13–26. Moses, upon advice, delegated responsibilities to leaders of tens, fifties, hundreds and thousands within the Jewish community^19,20^. Similarly, behaviour change campaigners – NGOs, governments and community workers - like Moses are overwhelmed with tasks and targets, and therefore need to empower others to create change impacts^19^.

**Fig. 1:**
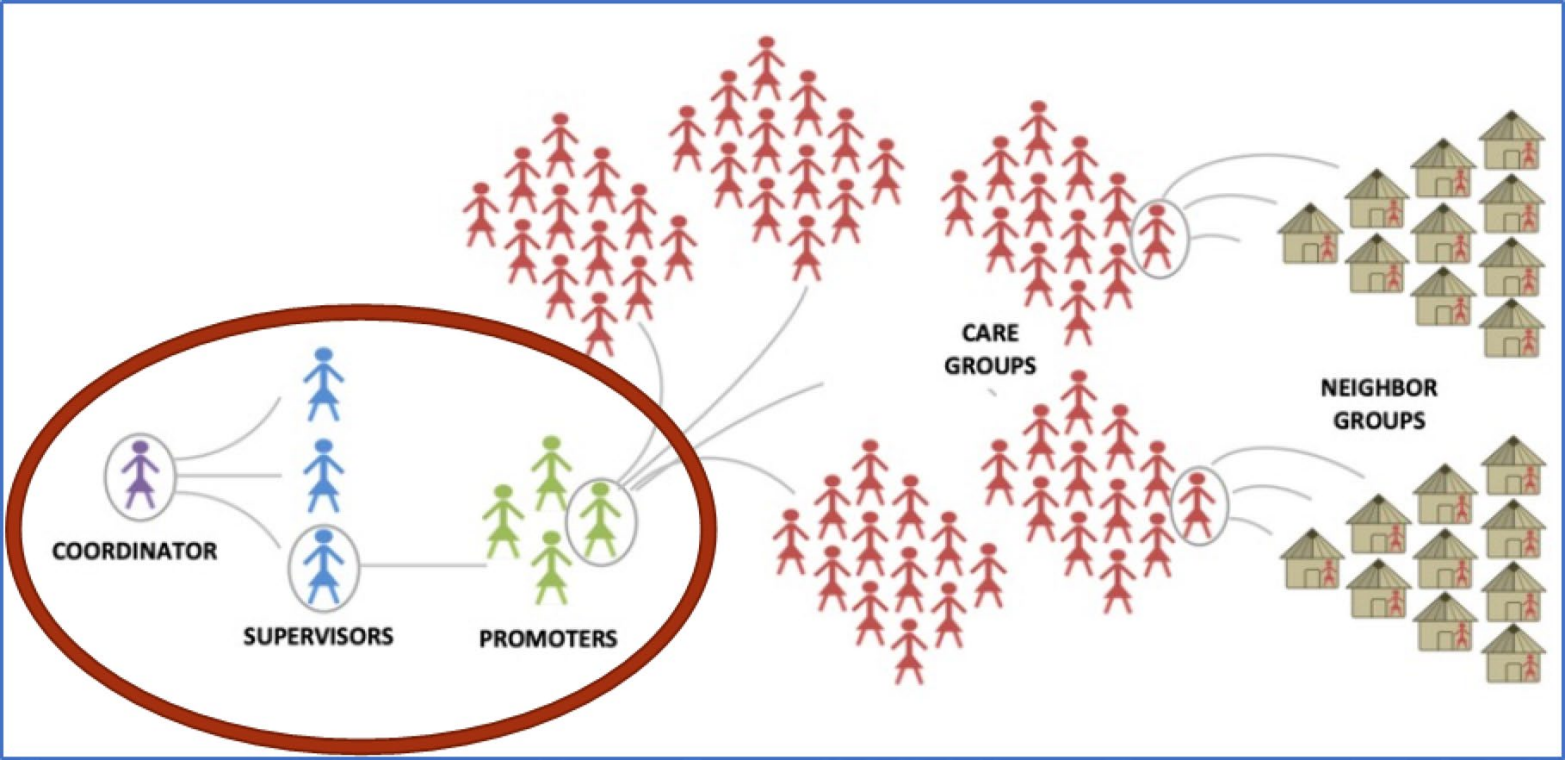
Nurturing Care Group model depicting cascading behaviour change and multiplying effect [Typically a coordinator, supervisors & promoters constitute World Vision/Government staff]^18^.

The approach is extensively implemented in at least 28 countries over the years, to necessitate its adoption to NCG for an integrated behaviour change intervention^19^. The NCG model launched by WV in Ghana comprises groups of 10 to 15 community-based volunteer behaviour change agents—referred to as leader mothers—who meet biweekly with project staff (e.g., coordinators, supervisors, and promoters) for training. These leader mothers then cascade behaviour change messages and activities to caregiver groups (mothers and pregnant women) within households and neighbourhoods^18^ (see Fig. 1). The intervention targeted building social support and cohesion among members while connecting neighbourhoods with community leaders and government services and personnel. Established linkages are to create a multiplying effect by facilitating the equitable reach of households with intervention messages through neighbour-to-neighbour contacts, home visits and group meetings^18,19^. Thus, NCG promotes behaviour change through peer support to establish new community norms for a healthy wellbeing.

The NCG intervention messages were on animal faecal management, household hygiene management, menstrual hygiene management, and child nutrition (including breastfeeding) in the two^2^ Programme Areas (APs) – Savelugu Municipal Assembly, and Sekyere East District Assembly. Trained health promoters delivered the WASH behavior-change messages to households via female caregivers, “leader mothers,” and neighborhood groups. Audiences were actively engaged using a pictorial manual with flip-chart images, songs, games, and stories to enhance message comprehension^21^. For a typical WASH behavior change intervention, households were sensitized to adopt individual initiatives and collective action around safe livestock husbandry and AFM in this case. The expected outcomes included household investment in livestock shelter, improved livestock care – feeding, watering, safe faeces removal through composting, secured penning to avoid straying, supervised grazing where feasible among others.

## Study area description and methodology

### Study area description and context

The study was carried out in two District Assemblies, namely Sekyere East and Savelugu Municipal which are World Vision Programme Areas that received the Nurturing Care Group intervention. The intervention was implemented in 41 communities between June 2019 and December 2020. Project communities were among the deprived and hard-to-reach areas in the districts^21^.

### Sekyere East District Assembly

The district is among the 43 Metropolitan, Municipal and District Assemblies (MMDAs) in the Ashanti region, with its district capital as Effiduase. The population of the entire district is 74,789, hosted by about 20,000 households with an average household size of 3.5 and occupying a total land surface area of 240 Km^2^^22^. Like the regional population structure, there are more females (52%) than males (48%) and urbanization is around 62%, almost equal to the regional figure of 61.6%^22,23^. Majority of the people (59%) are in the active age group 15–64 years^23^.

### Savelugu Municipal Assembly

The assembly was part of the erstwhile Savelugu-Nanton Municipal Assembly and following its cleavage it is among the 16 MMDAs forming the new northern region, and Savelugu township is its district capital^24,25^. The municipality with a total land surface area of 1,550 Km^2^ has a population of 122,888 with annual growth rate of 2.6% based on the recent 2021 Population and Housing Census (PHC)^26^. The female population is in a slight majority of about 51% and most of the people (53%) are in active age group of 15–64 years^26^. The municipality has around 23,000 households with an average household size equal to the regional value of 5.2 but more urbanized (63%) than the entire region (45%)^22,26^. The rural representation is only 37% of all settlements^22^ likely because of rapid urbanization from the regional capital Tamale.

### Study design and data collection

The approach adopted was cross-sectional qualitative design involving selected communities that received the NCG intervention. The data collection campaign was carried out between November and December 2021 in 12 selected communities: 6 each from Savelugu Municipal and Sekyere East District Assemblies. In each study district, the six selected communities were three communities that the implementer (World Vision) other than the research team considered “successful or who did make change” and other three tagged “less successful or who did not make change” in animal faecal management post intervention. The details for calling communities “successful” and otherwise was not privy to this evaluation study. The qualitative data for the study was collected through focus group discussion (FGDs) and in-depth interviews (IDIs) with predominantly female adult participants (at least 18yrs and above) who included neighbours and care groups. Participants were recruited based on their availability at the time of the survey by random picking through a community leader and/ or a care group leader after a successful community entry and observing local protocols. A total of 12 FGDs and 20 IDIs were carried with FGD participants ranging 8–24 people. The large participant sizes for FGDs were sometimes allowed to generate a simultaneous wide range of experience, shared narrative and dialogue, and co-construction^27–30^ in attempt to achieve efficiency within time constraints with available participants and resources^30^. This was done using two experienced facilitators, and a translator to manage turn-taking, probes, curbing dominance and opportunity to affirm agreement/disagreement on key points^31,32^. The interviews lasted 25–55 min for FGDs and 10–16 min for IDIs.

FGD and IDI tools used to facilitate interviews were structured around key thematic areas: animal management background (kind of animals owned, penning, and feeding practices and wellbeing), experience and attitudes regarding animal faeces management, lesson recall, action taken and challenges. The interview sessions were conducted in the local languages of participants - Dagbani for Savelugu communities, and Twi for Sekyere East. For Savelugu, the research team worked with two experienced field and community development officers as translators. The translators were recruited with the support of World Vision Area Program Office. A one-day training session was conducted for the field team including the translators, to ensure quality control on the field. The training featured role plays and tool pre-testing to ensure shared understanding of key concepts, consistent facilitation, accurate translation, effective information capture (recording and field notes) and also clarified team roles and field coordination.

### Data management and analysis

Field notes were recorded in the English language translation during FGDs/IDI sessions. Audio recordings were also done during interview sessions to capture the discussions in the local dialects and English language translations. Transcriptions were done using Microsoft Office (Word) by adapting the recommended three-stage approach to minimize errors^33^. Thus, audio recordings were first listened to (without transcription) alongside cross-checking field notes for re-familiarization and quick identification of gaps in the written notes, followed by full transcription by filling in gaps identified, and then reviews (in turns by the two core researchers) to resolve any discrepancies^33^. The transcripts were transferred from Microsoft Word to Excel to create a database for analyses. The analysis involved identifying key- terms for trends and prevalence through an inductive, mixed method content analysis guided by the pre-structured themes in the FGDs and IDI tools^34,35^. Additionally, we considered the emergence and prevalence of topical responses when identifying key thematic findings. Verbatim statements – quotations from individuals or where appropriate, by group consensus – were used to substantiate the analysis^35^. Also, Microsoft Copilot (Microsoft Corporation, Redmond, WA, USA) was used for grammar check and style suggestions during manuscript revision and all edits were reviewed and approved by the authors.

### Ethics declarations

The research protocols used for the study were approved by the Joint Committee on Human Research, Publications and Ethics of Kwame Nkrumah University of Science and Technology (KNUST), School of Medical Sciences and the Komfo Anokye Teaching Hospital with the Ref: CHRPE/AP/418/21. We confirm that the research followed relevant guidelines and regulations such as the Declaration of Helsinki including seeking of informed consent from participants after reading and explaining information about the study to them (in English and the local dialect translations). Verbal informed consents from participants and/or legal guardians were witnessed by the leaders who recruited participants, and the consent was documented as part of the field recordings (audio and field notes). Participants were informed of no direct benefits from participation in the study and that any information gathered was anonymised according to the approved research protocol.

### Limitations of the study

The study is limited in scope in terms of the number of communities - focusing on few successful and less successful intervention communities. The findings are based on qualitative study that relies on individual and group accounts of their experience and this could be associated with recall bias, satisficing, observer bias and the Hawthorne effect. The study relied on purposive sampling of participants who were available and willing to participate voluntarily in the data collection. Although the intervention employed a controlled before-and-after trial design aimed at improving key WASH related behaviors and management practices^21^, the current findings are exclusively based on post-intervention evaluation data.

## Results

### Study participants

Study participants were adults (18 years and above) and belonged to neighbourhood and/ or caregiver groups in the study communities. Almost all FGDs participants were women (151 females versus 4 males); the males came from two mixed groups in Sekyere East (Table 1). All 20 IDI participants were females. In total, 175 people took part in the study which targeted women, the primary recipients of the NCG intervention.

**Table 1:** Study participant numbers and gender.

Study areas	No. of communities	Number and gender of participants	
		FGDs, participants (range)	IDIs
Savelugu	6	6 female groups, 65 (8–19)	10 females
Sekyere east	6	4 female groups, 65 females (8–24) 2 mixed groups, 3 males & 10 females 1 male & 11 females	10 females
Total	12	12 (151 females with 4 males)	20 females

### Domestic animal types and benefits

Domestic animals reared in the study communities were predominantly livestock and pets (Table 2). Livestock comprised cattle, goats, sheep, fowls, rabbits, pigs, and donkeys, while the pets were cats, dogs, and doves. Communities in Savelugu kept both a larger number and greater variety of animals than from Sekyere East. In Savelugu, only single community reared pigs linking the observation to its predominant Islamic communities.

Most participants kept poultry, 62 and 67 households respectively for Sekyere East and Savelugu, followed by goats (33 and 47), then sheep (43 in Savelugu only), cats (22 and 15), and others (Table 2). Participants reported deriving multifaceted benefits from the reared animals including income, animal-source nutrition, compost for crop production, and socio-cultural functions and rituals.

**Table 2:** Animal types reared in communities and key benefits derived from the animals.

Study area	Types of animals owned		Key benefits derived from animals
	Community-wide	Number of participants owning specific animals	
Savelugu municipal assembly	Cattle, Goat, Cats, Dogs, Sheep, Fowls (guinea fowl, ducks, chicken, dove), Rabbit, Pigs^**1**^, and Donkeys.	Fowls (67); Goat (47); Sheep (43); Cats (22); Cattle (19), others (8)	✓ Sold for cash for feeding, kids school fees, and support family upkeep. ✓ Meat for food/nutritional supplement for the family. ✓ Animal droppings/faeces with household organic waste are used as manure on the farm to support crops. ✓ Use animals for religious activities (e.g., sacrifice, festive celebrations etc.). ✓ Dinner for special occasions /celebrations/hosting special guests.
Sekyere east district	Cattle, Cats, Dogs, Goat, Sheep, Fowls (chicken), and Pigs.	Fowls (62); Goat (32); Cats (15); Sheep (12); Dog (9), Cattle (1).	All points raised at Savelugu IN ADDITION to the following: ✓ Dogs and cats provide security (including controlling pests and rodents) and used for hunting. ✓ Animals as presents/gifts to loved ones during festivities/occasions.

^1^Only in one place, a non-Islamic community.

Verbatim quotes from FGDs and IDIs on benefits from livestock included:

“…*generate income to support household expenses …; livestock droppings use as manure for farming – this boosts crop harvest; meat for nutrition for family diet and special dishes for occasions and hosting guests; and also livestock for religious sacrifices.” (FGD, successful community Nakpanzoo – Savelugu, November 24, 2021).*

“*We generate income from livestock sales for household and children’s educational expenses including medical bills. Livestock droppings are used as manure for farming. Livestock meat is served on occasions/seasons and hosting of special guests.*” (FGD, less successful community Mogla – Savelugu, November 24, 2021).

“*Some sell livestock for income to cater for children’s basic needs*,* meat as a source of nutrition*,* we present them as gifts to other people*,* dogs are used for security and hunting*,* and cats fight pests and rodents.”* (FGD, less successful community Asukorkor – Sekyere East, December 8, 2021).

“*Meat for nutrition to support family diet*,* security from dogs and cats*,* selling livestock for income to pay children’s school fees and other expenses …… and meat for celebrations and festivities.”* (Mixed FGD, less successful community Nkwankwanua – Sekyere East, December 10, 2021).

“A widow, *I sell livestock to generate income for my family – feeding them*,* paying my sons’ school fees*,* and buying seedlings for the cropping season. I also use sheep for the Ramadan sacrifice (Muslim religious sacrifice)*,* and serve the meat during special occasions such as when we host visitors or celebrate naming ceremonies.”* (IDI 2, successful community Sandu – Savelugu, November 25, 2021).

“*Meat provides essential nutrition for the family diet. Sometimes I sell livestock to generate income for household expenses. Cats offer companionship and help control pests – preying on mice*,* lizards*,* snakes and other rodents.”* (IDI 1, less successful community Apemso – Sekyere East, December 9, 2021).

### Community approaches to keeping animals

Prior to the intervention, most animals were kept free-range; only a few owners combined free-range systems and penning or tethering. Animals were allowed to roam or scavenge during the day (especially in the dry season) and at night owners sheltered using methods such as:

- Keeping animals in pens/shelters.
- Tethering animals to poles/walls,
- Leaving animals in the courtyards, compounds of houses, and.
- Allowing animals to freely rest in neighbourhoods, streets for the night.

Specifically, the few sheltered livestock were released between 3:00 and 4:00 pm to scavenge and then returned to their pens at 6:00 pm.

Some quotes from participants are shared below to support our findings:

“*Some sheep and goats are allowed to roam freely during the day and are penned at night. Fowls have no fixed shelter; they perch in nearby trees overnight and forage freely during the day. Cattle are penned at night and taken by Fulani herdsmen to graze in the field during the day. Donkeys*,* which have no shelter*,* carry loads by day and are tethered to trees at night.*” (FGD, less successful community Gbumgbum – Savelugu, November 23, 2021).

“*Few of us have shelters for our livestock and others don’t have which encourages stray animals in the community with associated nuisance like littering with faeces. Some of us with shelters open them at 4:00 pm scavenge and lock them back at 6:00 pm.”* (Mixed FGD, less successful community Senchi – Sekyere East, December 10, 2021).

*“The entire community allows our livestock to scavenge especially around this time of the year when it’s hot*,* dry and feeding is expensive. We water them in the mornings and evenings when they are around. Some people herd their livestock home to their pens in the evening – especially to be watered and safe keeping against theft.”* (FGD, less successful community Kpachelo – Savelugu, November 23, 2021).

“*… livestock roam and lie around the community during dry seasons. In the rainy season we keep them in shelters where construction wood is readily available. Sheep and goats sleep in the open spaces or on the streets around houses. Typically*,* livestock are fed in the morning and are left to scavenge for the rest of the day. Fowls (chicken and guinea fowl) have no specific shelter*,* they perch in nearby trees at night and forage freely during the day. Cattle are kept in pens at night*,* and during daytime herdsmen take them to the field for grazing. For pigs have no shelter and must scavenger especially during the rainy seasons.”* (FGD, successful community Sandu – Savelugu, November 25, 2021).

*“…community practice mixed system – some members have shelter for their livestock and feed them*,* and others leave their animals to scavenge day and night. Those with shelter and/ or herdsmen take proper care of their livestock especially for cattle*,* goats*,* and sheep.”* (FGD, successful community Zieng – Savelugu, November 25, 2021).

*“The law from the district assembly requires that livestock may be released to scavenge at 4:00 pm and must be penned by 6:00 pm*,* but enforcement has lapsed. Although many owners now build shelters to avoid stray-animal surcharges this measure has proven ineffective. Owners argue that animals feed poorly in these shelters because of exposure to faeces and other unsanitary conditions.”* (FGD, less successful community Asukorkor – Sekyere East, December 8, 2021).

*“Bylaws on stray animals from the district assembly are supported by the unit committee and opinion leaders*,* yet they are ineffective. The problem in this community is inability to sustain good initiatives for the needed impact*.” (FGD, successful community Brofoyedru – Sekyere East, December 9, 2021).

*“Livestock roam on the streets in this community day and night because very few have shelters. Most owners are irresponsible with the management of their livestock.”* (FDG, less successful community Apemso – Sekyere East, December 9, 2021).

After the NCG intervention, communities improved livestock-management system with more penning alongside free‐range grazing. More owners penned their animals, while others allowed them to roam freely to forage. Even among those who penned livestock, animals were routinely released during the day to scavenge. Penning practices showed no variation between Sekyere East and Savelugu, nor between communities deemed successful and less successful.

Unlike Savelugu, communities in Sekyere East demonstrated high awareness of existing bylaws on stray animals. The communities had local arrangements – primarily through unit committees – to enforce regulations. Although the intervention initially inspired strong compliance, this momentum was not be sustained over time.

The caretakers of livestock in communities were mixed, mostly adults and sometimes children. The adults were females (mothers and/ or wives), males (fathers and/ or husbands) and children were the sons. Caretakers mainly fed and watered the livestock and sometimes cleaned the shelters/pens. However, the cleaning of the pens were mainly getting rid of animal faeces and this chore was done out by female caretakers – as part of their household sanitation and hygiene duties.

Some quotes from IDI respondents (females only) are shared below:

“*Feeding is done by my family members. The dog*,* fowls and cat are fed at the house*,* while the goat is fed in its shelter*,* allowed to roam to forage during the day and penned at night*.” (IDI 1, less successful community Senchi – Seykere East, December 10, 2021).

“*It is mixed approach. We sometimes feed the animals especially in the early mornings and allow them to roam and later in the evening they return to be fed at home as well.*” (IDI 2, successful community Brofoyedru – Sekyere East, December 9, 2021).

“*The kids and I take care of the animals especially cleaning the shelter and feeding them*.” (IDI 1, successful community Zieng – Savelugu, November 25, 2021).

“*I myself as the mother and my son take care of the animals. My son takes charge in keeping the animals only when he is back from school on vacations.”* (IDI 1, less successful community Gbumgbum – Savelugu, November 23, 2021).

“*The children take care of the animals especially feeding the chicks. Sometimes we buy feed from the grinding mill sites …*” (IDI 1, successful community Sandu – Savelugu, November 25, 2021).

### Prior attempts and barriers to AFM before intervention

The major prior attempts made by individual households concerning animal faecal management and their challenges (barriers) are summarized in Table 3. The main attempts had been providing temporary shelter for animals to minimize and/ or prevent straying and littering, and dumping animal faeces in bushes and at best directly on crops. Even animal faeces removal was irregular.

The main barrier to effective AFM (Table 3) across almost all communities included high cost of providing standard pens/shelter for animals. This led to overreliance on temporary structures when cheap materials became available during rainy seasons (especially for Savelugu communities). Also, there were perceptions regular cleaning of livestock shelters or removal of faeces were not critical. Lastly, little was known that other animal faeces (not only fowl droppings) could be used as manure (organic fertilizer) for crop production.

**Table 3:** Prior attempts and barriers to animal faecal management.

Areas	Prior attempts	Barriers
Savelugu	✓ Sometimes sweeping animal faeces/droppings and throwing them away and/ or with refuse at dump sites. ✓ Raise temporary shelters for some animals.	✓ No/little incentive to be cleaning animal faeces every day. ✓ Temporary shelters only possible at rainy seasons when shrubs/ tress/sticks become available as construction materials.
Sekyere East	✓ Sweeping animal faeces and throwing them away (anywhere in bushes). ✓ Sweeping and disposing AF into nearby farms/gardens. ✓ Sweeping animal faeces from shelter at most twice a week.	✓ Did not see the need to clean animal faeces each day. ✓ Did not know nor valued all animal faeces as fertilizer for crop farms. ✓ Knew only fowl droppings as probable organic manure for crops.

Below are selected verbatim quotes from female participants:

*“We were used to leaving the faeces within our compound for a long time*,* making our environment dirty. Now we know the value of livestock faeces – it is used as manure on our farms.”* (IDI 1, less successful community Kpachelo – Savelugu, November 23, 2021).

*“…we were applying livestock faeces on our farms without a clearer understanding of the full benefits. From the intervention*,* we have learnt to apply properly livestock faeces as manure to reap full benefits.”* (IDI 1, successful community Nakpanzo – Savelugu, November 24, 2021).

*“… we used to throw animal faeces into the environment which made our surroundings unsanitary with odour nuisance. Now we do co-composting with food waste to produce manure for application on our farms while keeping our neighbourhoods clean.”* (IDI 2, successful community Sandu – Savelugu, November 25, 2021).

*“We used to buy droppings from poultry farms for our crop farms but now we use our livestock faeces and the benefits are comparable to the ones we outsourced.”* (IDI 2, less successful community Apemso – Sekyere East, December 9, 2021).

*“I used to apply directly livestock faeces to crops but I have learned from the intervention that it is best applied when allowed to dry or kept for some days. I have also noticed an improvement in the yields of crops from my garden.”* (IDI 1, less successful community Asukorkor – Sekyere East, December 8, 2021).

“*Previously my livestock shelter was swept twice a week but nowadays because of the lessons from the intervention*,* I clean the shelter frequently and our compound is very neat.”* (IDI 2, less successful community Senchi – Sekyere East, December 10, 2021).

### Effectiveness and novelty of the AFM intervention

**Fig. 2:**
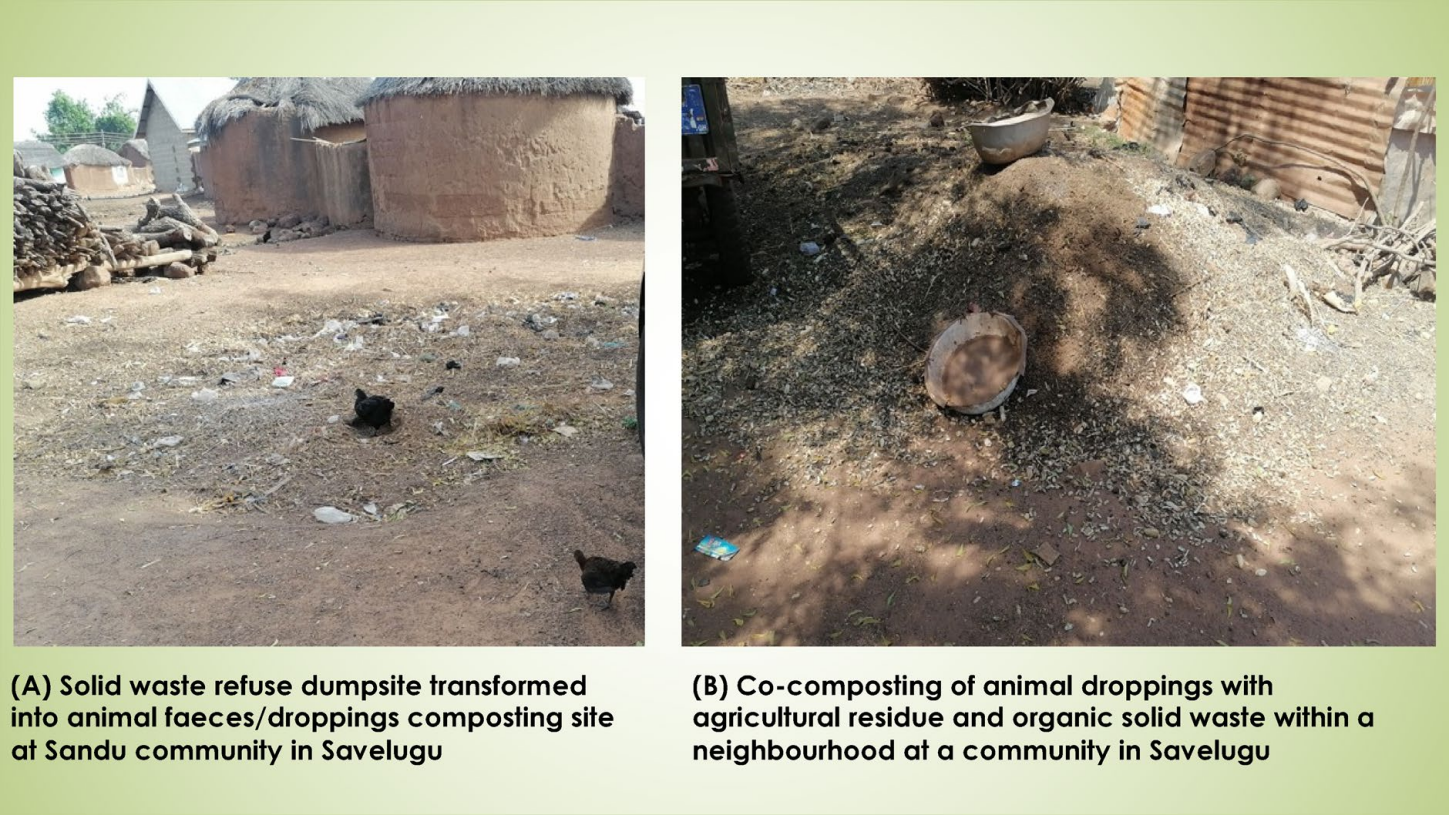
Composting/co-composting of animal faeces with organic waste in some communities (Savelugu).

Participants perceived the intervention to be effective through individual and community responses to project messages and lessons. Participants reported being inspired to adopt composting and/ or co-composting livestock faeces with organic waste to produce manure, constructed shelters or pens for livestock (although mostly temporary structures), attempted to secure veterinary services among others (see Table 4; Fig. 2). Beyond these actions, Sekyere East communities uniquely involved local leaders to support enforcement of bylaws and community resolutions towards effective AFM.

**Table 4:** Effectiveness of intervention felt in the study areas (based on FGD & IDIs).

Analyses	Study areas	
	Savelugu communities	Sekyere East communities
Differences	✓ Physical and practical evidence of implemented project lessons included co-composting of animal faeces with organic waste within neighbourhood/ compounds, raising of shelters including temporary ones, taking animals out for grazing, watering/feeding animals etc.	✓ Participants reported practicing composting with animal faeces as manure for their crop farms. ✓ Opinion leaders to some extent were used in enforcing local bylaws and community resolutions in the early stages of the intervention.
Similarities	✓ Animal management systems: Mixed, some households had constructed shelters, some fed their animals and others allowed them to roam and scavenge. ✓ Domestic animals including livestock were still roaming in the streets and neighbourhoods of all the communities visited. ✓ Most participants reported the use of animal faeces as manure on farms. ✓ Participants had difficulty in securing veterinary support services.	

Also, the intervention introduced innovative drivers to improve hygienic practices (Table 5). These included mobilizing women for collective action by emphasizing their roles as custodians of home management, incentivizing cost savings on commercial fertilizers via co-composting livestock faeces, and in Sekyere East AFM was linked to safe childcare and breastfeeding support in addition to engaging local governance structures to promote leadership.

**Table 5:** Novel aspects of the NCG intervention influencing effectiveness.

Study areas	Specific novel aspects of intervention
Both Savelugu & Sekyere East Districts	- Targeting of women was well appreciated, ^1^because women are responsible for the management of WASH and related issues in Ghanaian homes. - Adding value to animal faeces by composting which was a “premium” commodity in the intervention communities, being rural peasant farmers. - Linking AFM to the entire household environmental sanitation and hygiene practices - toilet ownership, kitchen and food hygiene, safe water withdrawal and storage, and safe childcare including breastfeeding.
Only Sekyere East District	- Linking intervention to safe breastfeeding and child nutrition. - Stimulated the involvement and influence of opinion leaders and community level political figures like unit committees, assembly members, and sometimes local government authority (District Administration or Assembly) with enforcement of communal and assembly bylaws.

^1^^34^.

All IDI participants (women) reported adopting AFM intervention lessons in their daily practices with some assertive responses:

*“Yes! I still apply livestock faeces as manure on the farm.”* (IDI 1, less successful community Kpachelo – Savelugu, November 23, 2021).

*“Yes*,* I still do the cleaning and occasionally consult the project booklets*,* which contain illustrations and materials that I often reference.”* (IDI 1, successful community Sandu – Savelugu, November 25, 2021).

*“Yes*,* I still do the cleaning of the livestock shelter and take the gathered faeces to apply on the farm.”* (IDI 1, less successful community Zieng – Savelugu, November 25, 2021).

*“Yes*,* the project has been very helpful*,* and I am doing all that I can although I am not quite perfect at it yet.”* (IDI 1, successful community Brofoyedru – Sekyere East, December 9, 2021).

*“My shelter was recognized at the community information centre as the best*,* and many people came to see it and improved theirs. The intervention helped us to secure financial support from a credit union. Yes*,* I continue to apply what I learned and I have my peace now.”* (IDI 1, less successful community Senchi – Sekyere East, December 10, 2021).

*“Yes*,* I continue to practice the lessons learned from the project and it has prevented me from quarrelling with neighbours because my livestock are restrained from spoiling people’s properties.”* (IDI 2, less successful community Senchi – Sekyere East, December 10, 2021).

### Level of satisfaction with AFM in community post NCG intervention

FGDs revealed widespread dissatisfaction with current AFM across study areas (Table 6). In Savelugu, 4 out of 6 groups described themselves as “somewhat satisfied” reflecting low approval of the current situation – far below their expectation. In Sekyere East, 5 of 6 FGDs registered clear dissatisfaction. Successful and less successful communities showed no notable differences in FGDs responses.

In contrast, IDIs painted a more positive picture, especially in Savelugu, where 8 of 10 respondents reported satisfaction with AFM. In Sekyere East, only 2 respondents were fully satisfied and four were “somewhat satisfied” (Table 6).

**Table 6:** Satisfaction with present animal management in community.

Study Areas	Communities	Level of satisfaction	Study Participant Group	
			FGD (consensus)	IDIs
Savelugu District	Successful (3)	Yes, satisfied	0	3
		No, not satisfied	0	1
		^*^Somehow/somewhat satisfied	3	1
	Less successful (3)	Yes, satisfied	1	5
		No, not satisfied	1	0
		^*^Somehow/somewhat satisfied	1	0
	All (6)	Yes, satisfied	1	8
		No, not satisfied	1	1
		^*^Somehow/somewhat satisfied	4	1
Sekyere East District	Successful (3)	Yes, satisfied	0	0
		No, not satisfied	3	4
		^*^Somehow/somewhat satisfied	0	1
	Less successful	Yes, satisfied	0	2
		No, not satisfied	2	2
		^*^Somehow/somewhat satisfied	1	1
	All (6)	Yes, satisfied	0	2
		No, not satisfied	5	4
		^*^Somehow/somewhat satisfied	1	4

^*^Included split decision where some members were satisfied, and others were not; otherwise largely by consensus

Some notable responses from participants were recorded below:

“*Yes*,* we are satisfied to some extent because we now know the number of animals we have and do keep them well. Also*,* no because some livestock still stray*,* get lost*,* and some are unable to raise shelter for the animals.”* (FGD, successful community Sandu – Savelugu, November 25, 2021).

One woman, met with audible disapproval from the rest of her group members, remarked:

*“… our husbands should provide - or aat least help build shelters*,* but they usually claim they have no time and offer excuse after excuse. We also struggle to feed and water the animals in the dry season*,* so it’s cheaper to let them to scavenge and fend for themeslves.”* (A participant of FGD, Sandu successful community – Savelugu, November 25, 2021).

*“We are not adequately satisfied because most of us cannot afford to build shelters for the animals*,* so they still scavenge and defecate everywhere. Feeding livestock is expensive in dry season*,* therefore we allow them (animals) to forage on their own in the fields and bushes.”* (FGD, less successful community Mogla – Savelugu, November 24, 2021).

“*We are not satisfied because some animals are still roaming and defecating everywhere including destroying farms… Everyone here must raise animal shelters and encourage others to have shelters for their farm animals.”* (FGD, less successful community Kpachelo – Savelugu, November 23, 2021).

“*Yes*,* we are satisfied! … our livestock look healthier than before.”* (FGD, less successful community Gbumgbum – Savelugu, November 23, 2021).

“*We are noot satisfied because most livestock stray including onto roads and are sometimes knocked down by cars and motorbikes. Owners need to have shelters to pen their livestock especially at night*.” (IDI 1, successful community Zieng – Savelugu, November 25, 2021).

“*We are not ok! Animals still roam the streets. Community leaders should work with the District Assembly to enforce safe domestic animals management while we continue to apply lessons from the project. Effective enforcement of laws in the community is critical for advancing AFM.*” (IDI 2, less successful community Apemso – Sekyere East, December 9, 2021).

“*No*,* we are not satisfied*,* most people don’t apply the project’s lessons that we have learnt so far. Unit committee’s enforcement of laws on stray animals is imperative for sustainable AFM post intervention. Continuous commitment from the committee is needed.”* (IDI 1, successful community Nkwankwanua – Sekyere East, December 10, 2021).

*“I will rate my satisfaction at 50% because broken shelters are not repaired nor rebuilt. Reminder visits are needed and the Assemblyman or District Assembly should begin catching stray animals on the streets.”* (IDI 2, successful community Nkwankwanua – Sekyere East, December 9, 2021).

***Potential challenges and barriers to NCG intervention in communities***

The barriers to progress and impact (see Table 7) of the NCG intervention were identified under four themes:

- Leadership and gender roles: roles of men/husbands as household decision-makers were unclear or marginalized, limiting buy-in and shared responsibility.
- Institutional gaps in conflict resolution: no formal channels existed to manage disputes, existing community- or district-level arrangements were inadequate including weak enforcement of bylaws.
- Technical and resource constraints: communities had insufficient local skills and technology to build low-cost animal shelters and to produce sustainable year-round livestock feed.
- Sustainability of behaviour-change support: no or weak institutionalized system for ongoing sensitization and education to reinforce positive behaviours towards improved hygiene and livestock management.

**Table 7:** Challenges or barriers to the intervention identified through analysis of FGDs & IDIs.

Study area	Main barriers requiring attention
Savelugu communities	✓ Role of men/husbands as family heads who lead decision-making and implementation of actions in the home were unclear nor felt in the project. ✓ Lack of institutionalised systems or channels for conflict resolution and management on disputes concerning reared animals. ✓ Lack of local technical understanding and skills to provide appropriate low-cost shelter for animals. ✓ Lack of local skills and technology to produce sustainable feed/foliage for animals all year round including dry seasons. ✓ Lack of local systems to drive long-term impact of behaviour change through continuous sensitization and education.
Sekyere East communities	✓ Lack of robust and sustainable institutionalised laws and enforcement systems to incentivise proper animal management practices. ✓ Inadequate and/ or malfunctioning existing arrangements (at community & district levels) for conflict resolution on disputes related to reared animals. ✓ Household sanitation (toilet provision) was introduced as part of the intervention, but inadequate information and support were shared with beneficiary communities. E.g., Sanitation marketing (SaniMart) as part of interventions could promote uptake of household toilet provision as a complementarity to other safe hygiene promotions. ✓ Almost all men/husbands were excluded in the project implementation except very few represented as care group members in only two communities.

Some verbatim quotes from study participants are shared below:

*“We were told to construct improved toilet facilities*,* but there is no money. We sometimes find it challenging to get clean water to water our livestock especially during the dry seasons. The cost of providing shelter for livestock is a challenge. …our husbands need to support our effort especially for safe animal faeces management.”* (FGD, successful community Sandu – Savelugu).

“*… provision of improved household toilets is expensive and unless the men (our husbands) decide to prioritize and build them*,* we women can only ask and nothing will happen.*” (FGD, successful community Nakpanzoo – Savelugu).

*“… household toilet ownership is still a challenge although some of us now have toilets in our compounds.”* (FGD, less successful community Kpachelo – Savelugu).

*“… keeping livestock in shelter affects birth rates and also decreases their weight so we prefer that they scavenge for forage in the field.”* (FGD, less successful community Apemso – Sekyere East).

*“The shelter is difficult to raise because of the cost involved – especially construction materials like roofing sheets*,* nails*,* wood etc.”* (FGD, less successful community Asukorkor – Sekyere East).

*“Getting a shelter to pen the livestock has been challenging because of lack of money to buy the construction materials.”* (FGD, successful community Feyiase – Sekyere East).

*“*Efforts to provide animal shelters did not continue effectively, so animals are now seen roaming the streets due to weak law enforcement.*”* (Mixed FGD, less successful community Nkwankwanua – Sekyere East).

“*Education on proper animal husbandry and AFM should continue with emphasis on the benefits. Some individuals still neglect animal welfare focusing solely on the personal gain. Furthermore*,* enforcing laws that promote effective animal rearing would be essential for community improvement.*” (IDI 1, successful community Zieng – Savelugu, November 25, 2021).

“*We need access to funds to support improved livestock rearing especially for constructing shelter/pens*,* and technical assistance*,* in addressing challenges including household sanitation (toilet provision)*.” (IDI 1, successful community Sandu – Savelugu, November 25, 2021).

“*Education on the use of livestock faeces on farms must be repeated with more clarity to address the perception that only fowl faeces can be applied on crops.”* (IDI 1, successful community Nkwankwaanua – Sekyere East, December 10, 2021).

*“…because poor management of livestock has caused much enmity among community members*,* all well-meaning citizens especially leaders like the Unit Committee should be concerned to maintain peace and order.”* (IDI 2, less successful community Senchi – Sekyere East, December 10, 2021).

One of the FGDs reported that a neighbouring community learned of their early success story with AFM and adopted mobilizing community effort to control stray livestock. The participants claimed that such spillover effect from the NCG intervention from their community is well patronized by their neighbours.

*“The commitment of community leaders is essential to ensuring effective enforcement of livestock-related laws and inspiring dedicated committees to oversee them*,* Akuakrom stands as a prime example – having learned from our early success story*,* they are now motivating other communities. Their progress should inspire us to return to the foundational days of the NCG project when we collectively excelled in controlling our livestock as a community.”* (FGD, successful community Brofoyedru – Sekyere East, December 9, 2021).

## Discussion

The intervention targeted female community members through volunteer leader mothers who engaged with other women, including young females and girls). The approach attempts to leverage women’s primary role in home management including sanitation and hygiene chores, as documented in other studies^34,36^, to enhance animal faeces management. Unlike the more gender-restricted implementation in Savelugu, the female-led initiative in Sekyere East adopted a relatively flexible approach that encouraged the participation of a few voluntary men from Nkwankwanua and Senchi communities. The male counterparts with their influence within the communities inspired the core women groups and their communities. This variant approach aligns with the assertion that males could be recruited for influential roles for effective implementation in female-led interventions^37^.

In our study communities, most animals reared were livestock—primarily fowl, goats, sheep, and cattle—in addition to pets such as cats and dogs. Livestock ownership among study participants reflects broader rural Ghanaian trends, with nearly all households rearing fowl and over half keeping goats. This aligns with documented patterns of ruminant and non-ruminant livestock being managed under extensive systems^38^. With their extensive free-range system, animals forage by day and are penned or tethered within compounds and yards each evening. Thus, extensive grazing remains the dominant husbandry method like in other rural parts of Ghana^39,40^. This is partly due to persistent perception that the approach offers some positive outcomes – that the animals exhibit some resilience with minimal health challenges as other studies report^38^.

To livestock owners, major benefits from reared animals include income to support household needs, improved nutrition, access to manure from animal faeces, and socio-cultural functions – presenting as gifts, use in festivals and religious rituals (e.g., sacrifices), as recorded in other studies^41^. These benefits are substantial contribtions to the socio-economic and cultural livelihoods of rural subsistent families^10,42^ especially during dry seasons when crop yields are low^38^. Beyond livestock, the few pets – particularly dogs and cats in Sekyere East – also provide functions like household security (e.g., control of stray animals and rodents) and hunting support. Some intangible and non-market benefits from livestock may play a role in fueling the perception that free-range system is economically viable for peasant households^10^.

Animal caregivers in these communities are primarily adults – mothers or wives and fathers or husbands with children, especially sons assisting sometimes. In few cases especially in Savelugu, cattle owners hire herdsmen to tend the livestock. These arrangements are common practices that are reported among livestock-rearing communities in Ghana^39,41^. Caregivers’ primary duties are consistent with records in literature – feeding and watering livestock and sometimes cleaning animal shelters or pens. Shelter cleaning (mostly faeces removal) is regarded as a domestic chore reserved for female household members, confirming women’s broader WASH responsibilities at home^34,43^.

Consistent demonstration of heightened awareness and immediate commitment to apply lessons emphasize the intervention’s ability to shift behaviours. For instance, the collective efforts to corral animals for improved AFM are a positive development. Like other WASH behaviour-change interventions, it is able to cause at least an immediate impact or project outcomes^44^. In Sekyere East, the commitment to enforce district assembly bylaws on stray animals is a demonstration of impact. This aligns with studies that sanitation and hygiene interventions should involve bylaws enforcement among other things^45^. The momentum for bylaws enforcement waned over time because of barriers similar to those recorded in literature – limited or no incentives for collective action, inadequate capacity to handle conflict resolutions, and inadequate support from the Assembly^45–47^.

Before the intervention, barriers to improve AFM included unsustainable penning methods (seasonal, temporary shelters), lack of motivation for regular and purposeful animal faeces removal, lack of resources and technical expertise. Penning presents its own barrier: livestock owners struggle to supply adequate feed without the animals scavenging – especially in the dry season when feed is both scarce and prohibitively expensive. Such challenges alongside the pervasive perception that scavenging benefits animal health and welfare are similarly reported among livestock farmers including Ghana^6,48^. Farmers also contend with prohibitive cost of veterinary services and other financial constraints which are confirmed in other studies that such could limit herd size^38,39,41^. Moreso, inadequate access to professional veterinary services could contribute to antibiotic abuse among livestock owners – this comes with the challenge of antibiotics and antimicrobial resistance, a One Health issue^49^.

The novelty of the intervention is linked to the outcomes and impacts which align with the One Health approach to addressing zoonotic risks. The intervention attempts to satisfy the defined core elements of One Health – animal, human, environmental, and governance domains^6,48^. Further to that, the gender-responsive approach leverages linking women as home managers to AFM. Also, incentivizing composting/co-composting offers a dual benefit: reducing household expenditure on commercial fertilizers and boosting crop yields via self-supplied organic manure. This is context-sensitive approach to improving rural livelihoods through the use of available local resources^50^. Linking the intervention to local concerns like safe childcare and breastfeeding, and deliberately involving opinion leaders, particularly in Sekyere East, enhances its local relevance. Local level governance engagement strengthens community ownership and lend legal weight to collective decisions initiated by interventions. Thus, corroborating evidence that effective project interventions must be intentional in embedding local leadership structures for normative shift^37,50^.

The limited engagement of men is a weakness in the intervention design and implementation phases. In male-headed households and communities, where socio-economic decisions often fall to men, their exclusion or limited involvement constrains intervention successes. Evidence suggests that even female who own livestock maintain deference to husbands or male heads due to prevailing household hierarchies^51^. Unlike urban contexts, where individual ownership is more common, rural livestock ownership tends to be household-based, with men exercising greater control as the family head^41^. Although interventions promoting full women participation could be up to seven times more effective in water and sanitation outcomes^52^, such a success could be undermined when male counterparts are excluded – because they are the key decision-makers in patriarchal homes and communities on most matters including livestock rearing^37,53^.

The widespread dissatisfaction with the state of AFM in communities – following the erosion of earlier gains and the lowering of standards which once instilled pride in their collective efforts – testifies the need to integrate sustainability into rural interventions. The inability to sustain immediate intervention gains reflects a typical challenge in behaviour change interventions – namely slippage, where motivation declines following the withdrawal of project implementers^44^. Slippage underscores the fragility of progress when community engagement to use governance structures is weak or lacking especially when people report feelings of shame and discouragement – highlighting unmet expectations. In Sekyere East particulary, the initial momentum driven by intentional community involvement could not be maintained, likely due to inadequate local leadership capacity – an issue echoed by Tsekleves et al.^50^.

A number of design challenges in the NCG intervention cannot be overlooked. Notably, failure to actively engage men - particularly household heads, since they are the known decision-making authority in the home^54,55^. Again, weak local institutions hindered enforcement of bylaws and community resolutions – these form a key foundation for collective actions aimed at safe livestock rearing. Law enforcement is constrained by factors such as political interference, public criticism, lack of incentives, unrealistic expectations and lack of funding. These contribute to a non-responsive governance environment and inability to resolve common disputes such as animal theft, stray livestock damage, or ownership conflicts. These constraints corroborate reported challenges that characterize Ghana’s local governance regime^45,56–58^. Furtherance to that, the intervention lacked adequate technical backstopping and local financing mechanisms to support livestock owners. These bother on gaps in providing shelters, feedstock acquisition and storage (especially during the dry season), veterinary services, and access to appropriate technologies. Intervention design failed in addressing these gaps which are known challenges among livestock farmers or owners including those in Ghana^39^. Linking AFM to improved household toilet ownership and use could not translate into the expected outcomes like toilet constructions. This is likely due to lack of specific strategies targeting toilet uptake and even at scale – including sanitation marketing, which is a key component of a transformative WASH behaviour change interventions^59^.

Notwithstanding the challenges outlined, several strategic improvements could strengthen future AFM intervention design and implementation:

(i) There is a pressing need for strategies that employ continuous awareness creation, sensitization and education to promote and sustain safe animal husbandry including AFM. Extended timelines and adaptive strategies are necessary to achieve lasting impact because behaviour change interventions often unfold within complex socio-cultural contexts^60,61^. Given the likelihood of regression to previous practices – especially when beneficiaries experience fatigue or diminished motivation over time^44^, it is imperative that the roles of District Assemblies are actively integrated into both the design and implementation phases. Such involvement through either their Environmental Health and Community Development Units should extend beyond technical support to include ownership of post-intervention backstopping, coordination and supervision to ensure sustainability at scale.
(ii) The communities need technical support and innovative local financing mechanisms (e.g., crowd funding, soft loans, cooperative credit systems) to translate some of the intervention ideals into sustainable actions. Most livestock owners lack the ability to embark on initiatives such as mobilizing financial resources to construct standard shelters or pens, procuring feedstock, and even accessing technical expertise. Addressing these gaps calls for the active engagement and empowerment of relevant local institutions to provide the needed support. For instance, a community-managed fodder bank could be established to provide strategic feed reserves against drought periods, while banking institutions – farmer associations partnerships could facilitate tailored financial products for livestock rearing^39^.
(iii) Given that AFM forms part of sanitation and related issues governed by assembly bylaws^14^, enough attention should be given to strengthening frontline local governance structures for effective enforcement^45,57^. For instance, the delegated authority of unit committees should be actively supported by District Assemblies to enable them lead and /or co-supervise enforcement of relevant laws and community resolutions.

## Conclusion and recommendations for practice

Animals reared in study communities are livestock such as fowls, goat, sheep, and cattle including pets (e.g., cats, dogs, dove). The multifaceted motivations for keeping livestock among participants include economic, nutritional and socio-cultural benefits such as income, food security, ritual functions, and companionship. Communities recognize AFM not only as environmental and public health issue but also as an opportunity to produce organic manure – linking livestock rearing with improved sanitation and crop yield. Though the NCG intervention heightened awareness particularly around livestock penning, livestock faeces composting and environmental sanitation and hygiene nexus, practical barriers persist in beneficiary communities. Such constraints include seasonal feed shortages, prohibitive cost of constructing standard shelters or pens, limited access to veterinary services, and gendered decision-making dynamics at the household level.

The awareness promoted constructive reflections on unmet expectations and lapses in project gains, opening up opportunities for District Assemblies to initiate renewed collective actions. Institutional engagement, particularly with local governance structures, emerges as a critical differentiator. The deliberate involvement of unit committees and opinion leaders in Sekyere East evidently bolstered enforcement of bylaws and community buy-in, contrasting Savelugu’s weaker local governance integration and a lack of local champion motivation factor.

It is recommended that future interventions should:

- deliberately institutionalize local leadership into project design and implementation phases by clearly defining roles for stakeholders (e.g., unit committees, opinion leaders, local champions, and District Assemblies), and even establishing accountability mechanisms (e.g., community scorecards).
- embed gender-sensitive strategies to include engagement with male household heads to secure buy-in for key socioeconomic decisions like investing in livestock shelter, feed procurement and other AFM decisions.
- offer practical technical and financial support solutions to match the needs of livestock owners. Such could include piloting low-cost local shelter or pen options, facilitating community seed and fodder banks, linking veterinary services to schedule routine checkups, and innovative local financing (e.g., small grants for women, crowd funding, soft loans, cooperative credit facilities).

## Data Availability

All the relevant data supporting the findings have been shared directly in the manuscript itself as much as possible. Moreover, for contractual reasons authors are unable to share the raw data, and we believe that a reasonable request on the raw data acquisition could be made by contacting the funder via Bismark Yao Norgbe at bnorgbe@WorldVision.org.
